# Capacity and selection in immersive visual working memory following naturalistic object disappearance

**DOI:** 10.1167/jov.23.8.9

**Published:** 2023-08-07

**Authors:** Babak Chawoush, Dejan Draschkow, Freek van Ede

**Affiliations:** 1 Institute for Brain and Behavior Amsterdam, Department of Experimental and Applied Psychology, Vrije Universiteit Amsterdam, Amsterdam, The Netherlands; 2 Department of Experimental Psychology, University of Oxford, United Kingdom; 3 Oxford Centre for Human Brain Activity, Wellcome Centre for Integrative Neuroimaging, Department of Psychiatry, University of Oxford, Oxford, United Kingdom; 4 Institute for Brain and Behavior Amsterdam, Department of Experimental and Applied Psychology, Vrije Universiteit Amsterdam, Amsterdam, The Netherlands

**Keywords:** visual working memory, selective attention, capacity, eye movements, virtual reality

## Abstract

Visual working memory—holding past visual information in mind for upcoming behavior—is commonly studied following the abrupt removal of visual objects from static two-dimensional (2D) displays. In everyday life, visual objects do not typically vanish from the environment in front of us. Rather, visual objects tend to enter working memory following self or object motion: disappearing from view gradually and changing the spatial relation between memoranda and observer. Here, we used virtual reality (VR) to investigate whether two classic findings from visual working memory research—a capacity of around three objects and the reliance on space for object selection—generalize to more naturalistic modes of object disappearance. Our static reference condition mimicked traditional laboratory tasks whereby visual objects were held static in front of the participant and removed from view abruptly. In our critical flow condition, the same visual objects flowed by participants, disappearing from view gradually and behind the observer. We considered visual working memory performance and capacity, as well as space-based mnemonic selection, indexed by directional biases in gaze. Despite vastly distinct modes of object disappearance and altered spatial relations between memoranda and observer, we found comparable capacity and comparable gaze signatures of space-based mnemonic selection. This finding reveals how classic findings from visual working memory research generalize to immersive situations with more naturalistic modes of object disappearance and with dynamic spatial relations between memoranda and observer.

## Introduction

**Figure 1.**
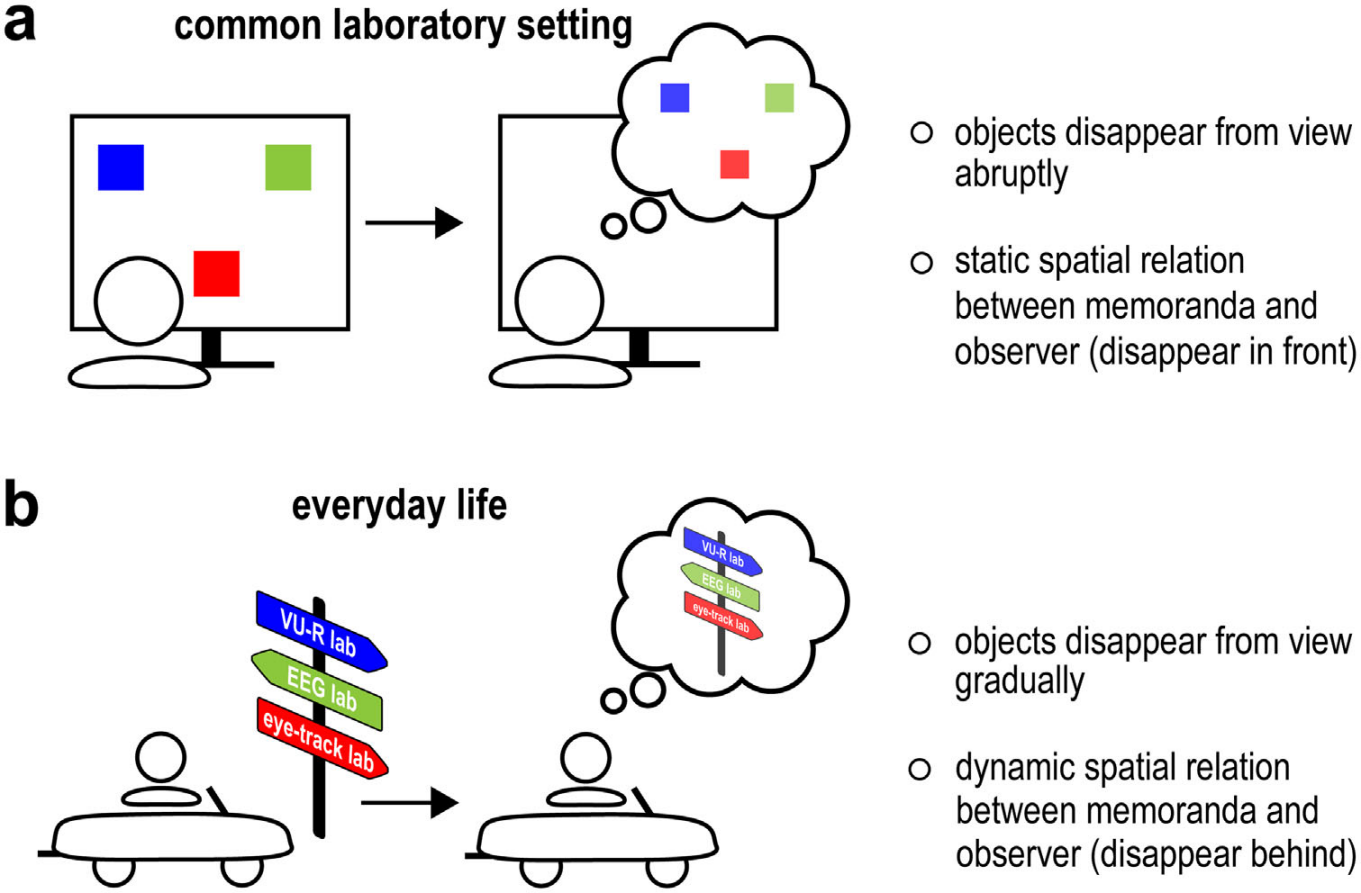
Visual working memory in common laboratory settings and everyday life. (**a**) Common laboratory setting in which visual objects disappear from view abruptly and in which spatial relation between observer and memoranda remain fixed. (**b**) Example of an everyday situation in which visual information disappears from view gradually and in which the spatial relation between observer and memoranda is dynamic, such as when objects disappear behind the observer.

Visual working memory pertains to the fundamental cognitive ability to hold available past visual information in mind to serve upcoming behavior (Baddeley, 1992; D'Esposito & Postle, 2015; van Ede & Nobre, 2023). In the psychological laboratory, visual working memory is commonly studied following the abrupt removal of visual objects from static two-dimensional (2D) displays (Figure 1a). However, in dynamic everyday situations, visual objects do not typically disappear abruptly from the environment in front of us. Rather, visual information tends to enter working memory after gradually disappearing from view, such as when driving by a road sign (Figure 1b).

It is important to understand whether classic findings from laboratory studies of visual working memory with static 2D displays generalize to such more naturalistic conditions. Not only is generalization a key challenge in contemporary psychology (Kristjánsson & Draschkow, 2021; Snow & Culham, 2021; Tatler & Land, 2011; Yarkoni, 2022), but it is also conceivable that working memory may operate differently in the two aforementioned conditions. First, although it is convenient, the process of abruptly removing visual objects in plain view has limited external validity (Draschkow, 2022) and may affect memory encoding and consolidation. Second, when seated behind a computer monitor from which visual objects are removed, the spatial relations between memoranda and observer remain fixed. In contrast, in everyday life, we often retain visual objects in working memory because the spatial relations between the memoranda and the observer are dynamic, such as following self and/or object motion. Our road sign example (Figure 1b) illustrates the point. We rely on working memory not because the road sign has ceased to exist in the external environment but rather because we drove past it. Here, the road sign is encoded while in front of us; however, once passed, the memorandum in question resides behind us.

Motivated by the above, we used VR to bypass traditional limitations of 2D displays while retaining high experimental control. VR enabled us to approximate the situation in which visual objects flow by participants, thereby gradually disappearing from view and changing the spatial relation between memoranda and observer (cf. Figure 1b). For reference, we also included a matched condition in which the same visual objects (in the same VR environment) were held static in front of the participant before being removed from view abruptly, mimicking more traditional laboratory tasks. This allowed us to form a bridge to traditional non-VR tasks before turning to our central flow condition of interest. We focused our investigation on two classic laboratory findings: (1) a capacity of around three objects, and (2) the reliance on space for mnemonic object selection.

### Visual working memory capacity

The capacity of visual working memory is limited. Although there is disagreement regarding the nature of this limitation (Brady, Konkle, & Alvarez, 2011; Ma, Husain, & Bays, 2014; Zhang & Luck, 2008), it is generally agreed that it is relatively easy to hold two visual objects in mind but considerably more difficult to retain four. This is consistent with estimates that visual working memory capacity plateaus at around three to four visual objects (Brady et al., 2011; Cowan, 2010; Luck & Vogel, 1997; Luck & Vogel, 2013; Vogel & Machizawa, 2004). At the same time, it has been demonstrated that the capacity of visual working memory may be larger when considering real-world (Brady, Störmer, & Alvarez, 2016) or more meaningful (Asp, Störmer, & Brady, 2021) objects. This suggests that capacity could potentially be higher under conditions that are more naturalistic; however, so far this line of inquiry has focused on the nature of the visual objects themselves. Here, we considered “mode of object disappearance” as another relevant dimension for understanding visual working memory under more naturalistic conditions, where visual objects disappear from view gradually in a more naturalistic manner versus abruptly in a more artificial manner.

### Space-based mnemonic selection

A second factor of interest was the use of memorized space for object selection in working memory. From studies with 2D displays—in which the spatial relation between memoranda and observer remains fixed (Figure 1a)—the role of space is well established. Space serves as a scaffold for working memory retention (de Vries & van Ede, 2023; Heuer & Rolfs, 2021a; Jiang, Olson, & Chun, 2000; Pertzov & Husain, 2014; Schneegans & Bays, 2017), as well as for the selection and prioritization of specific objects within working memory (Griffin & Nobre, 2003; Heuer & Rolfs, 2021a; Kuo, Rao, Lepsien, & Nobre, 2009; van Ede, Chekroud, & Nobre, 2019). One particular and recent demonstration of this involves spatial biases in gaze when selectively attending to objects in visual working memory (de Vries, Fejer, & van Ede, 2023; Draschkow, Kallmayer, & Nobre, 2022; van Ede, Board, & Nobre, 2020; van Ede, Chekroud, & Nobre, 2019; van Ede, Deden, & Nobre, 2021) in the absence of anything to look at (see also Ferreira, Apel, & Henderson, 2008; Martarelli, Ovalle Fresa, Popic, Globig, & Rothen, 2022; Spivey & Geng, 2001; Wynn, Shen, & Ryan, 2019).

An important open question, however, is whether memorized space is also used to select particular objects in working memory in more dynamic, everyday situations in which objects enter working memory after disappearing behind the observer. Critically, in such cases, the external locations associated with memorized objects are themselves out of view. We therefore asked whether we would still find the aforementioned spatial gaze biases as an index of space-based mnemonic selection.

## Methods

### Participants

Healthy adult individuals between 18 and 40 years of age with no history of psychiatric or neurological disorders and with normal or corrected-to-normal vision were recruited at the Vrije Universiteit Amsterdam. Sample size was set a priori to *n* = 25, based on prior studies that used comparable outcome variables (Draschkow, Nobre, & van Ede, 2022; van Ede, Chekroud, & Nobre, 2019; van Ede et al., 2020). Data from all 25 participants (age range, 18–32 years; 16 female; all right-handed) were retained for analysis. Participation occurred on a voluntary basis, upon informed consent and against a reward of course completion credits or compensation with €8 per hour. The experiment adhered to procedures approved by the local ethics committee of the faculty of behavioral and movement sciences at the Vrije Universiteit Amsterdam.

### Equipment, setting, and stimuli

The VR experience, eye tracking, and interaction were provided through the HTC VIVE Pro Eye head-mounted display (HMD, referred to hereafter as “goggles” for convenience) together with a hand-held controller (VIVE Developers, 2023a). The setup was powered by a Dell Alienware 15 laptop computer (Dell, Inc., Round Rock, TX) with Windows 11 (Microsoft, Redmond, WA). The experiment was programmed using Unity 2020.3.9f1 software, and eye-tracking measures were accessed using the VIVE Eye and Facial Tracking (VIVE Developers, 2023b) software development kit (SDK). The general interface to the VR equipment was provided by SteamVR and its corresponding SDK for Unity.

Wearing the goggles, participants were situated in a virtual environment—a lit light-gray room 10 (width) × 10 (length) × 5 (height) meters, in which all of the experimental conditions took place. Participants stood near the back wall (at a distance of ∼1 meter), facing the front wall (at a distance of ∼9 meters). A black fixation cross was painted on the front wall at a height of 20 cm below viewpoint. Stimuli appeared between the center of the room and the front wall. Participants provided input (task-relevant responses, as well as traversing the procedure in-between blocks) through the VIVE wireless controller, which they held in their right (dominant) hand and that appeared as a glove inside the simulation. Task-relevant interaction proceeded by means of moving/touching with the virtual hand; in between blocks, the trigger button (operated using the index finger) was used for confirming the start of blocks (and the eye-tracking calibration as part of it). Physics simulation, frame rendering, and eye tracking each occurred at 90 Hz. Further hardware specifications can be found on the HTC VIVE website (VIVE Developers, 2023a).

**Figure 2.**
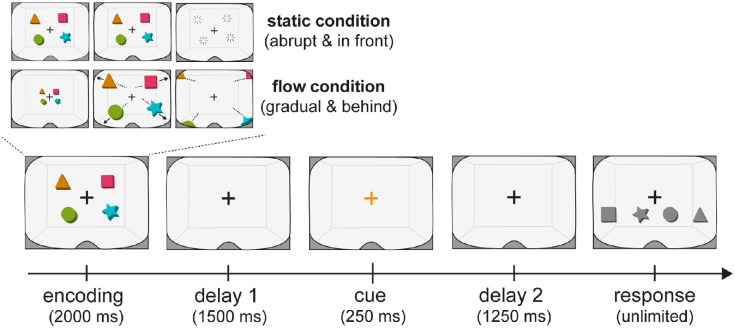
Immersive visual working memory task with manipulation of the mode of object disappearance. Participants performed a visual working memory task in virtual reality in which visual objects (colored 3D shapes) disappeared abruptly (static condition) or disappeared gradually behind the observer (flow condition). After a delay, a color cue prompted participants to select the color-matching shape from memory. After another delay, participants selected the cued shape among a series of colorless shapes by touching it with a virtual hand that was operated by the VR controller.

The task-relevant stimuli consisted of three-dimensional (3D) virtual objects and a fixation cross (Figure 2). Possible object shapes included a cube, sphere, cylinder, triangle, diamond, and star; possible colors of these objects were red, green, blue, magenta, cyan, and yellow. Any given trial contained either two or four objects, always in unique shapes and colors. Any presented object could occupy one of 10 configurational positions (“locations” for convenience) along an imaginary circle that was centered around the central fixation cross. Note that, in our critical flow condition (see Experimental design and procedure section), these configurational positions involved 10 linear trajectories along an imaginary “tube” where the locations varied while the overall configuration was preserved between the objects. Configurational object positions in degrees (0° pointing up) were 18°, 54°, 90°, 126°, 162°, 198°, 234°, 270°, 306°, and 342°; each slot was on the radius of 1 meter from the fixation cross (when projected on the plane perpendicular to the forward direction). In the static condition, this corresponded to a radius of approximately 14 degrees visual angle (DVA). In the flow condition (where the objects first appeared twice as far away), the objects started at approximately 7 DVA, reached 14 DVA midway, and ultimately disappeared from view at the maximal visible angle. Furthermore, for every occupied object slot on this memory array, the opposite slot was always occupied, as well, which provided an equal number of presented objects on the left and right halves of the array, respectively, as well as an equal number for the top and bottom halves. The objects of an always array appeared (and disappeared) simultaneously. During the task, the fixation cross remained at the same location.

### Experimental design and procedure

Participants performed a visual working-memory task in VR (Figure 2). The central manipulation (that was uniquely enabled by the use of VR) regarded the “mode of object disappearance” that could take either of two forms: flow or static. In our critical flow condition, objects traveled by the participant and therefore gradually disappeared from view behind the participant. For reference, we also included our static condition in which objects remained static in front of the participant and were removed abruptly (mimicking more traditional laboratory tasks with 2D displays, inside our 3D VR task). In addition to manipulating the mode of object disappearance, we manipulated memory load in both of the above conditions, such that a memory array of any given trial contained either two or four objects.

In the static condition the objects were presented 5 meters in front of the front wall (∼4 meters in front of the participant). The items always stayed in position for 2 seconds before being removed from view. In contrast, in the flow condition, the objects started 1 meter in front of the front wall (∼8 meters in front of the participant) and traveled through space in a linear trajectory toward the participant until ultimately disappearing behind the participant. Objects traveled at a speed of 4 m/s. Hence, in total, the time from object onset to disappearance was matched to the duration of object visibility in the static condition (2 seconds). Moreover, in the flow condition, the objects initially appeared at twice the distance from the participant compared to the static condition. In this way, the average visible position and, by approximation, the average displayed object size were also matched as closely as possible between static and flow conditions.

In every condition, participants performed the same cued recall task: Object disappearance was followed by a retention period of 1500 ms in which only the fixation cross remained visible. The retention period was followed by a cue in the form of a brief (250 ms) change in the color of the central fixation cross. The color cue prompted participants to select the color-matching object shape from memory. Then, 1500 ms after the cue, participants were presented with six randomly ordered black shapes (presented within reach and view, 10 cm below the fixation cross) from among which they were asked to select the cued memory shape by touching it. Upon touching one of the black shapes, participants were shown immediate feedback on the correctness of a response: The shape either shattered into pieces (correct) or dropped to the floor (incorrect). The feedback animation lasted 1000 ms.

Task instructions were provided at the start of a session, followed by a few practice trials to introduce the possible conditions. After practice, the experiment was structured into 10 blocks, each containing 40 trials in which the conditions were pseudorandomized, such that a block contained 10 trials for every of the four respective conditions. The total number of trials per participant was 400 (100 per condition). Eye-tracking was calibrated at the start of each block using the respective built-in VIVE solution. Each block took approximately 6 minutes, totaling 60 minutes. In between blocks, participants had the opportunity to take short self-paced breaks.

### Data analysis

All data were analyzed using MATLAB 2022a (MathWorks, Natick, MA), with the exception of the *d*′ sensitivity analysis, which was performed in R Studio (R Foundation for Statistical Computing, Vienna, Austria). For reference, we first considered the static condition that was included with the primary purpose to mimic more traditional laboratory studies of visual working memory using static 2D displays but here were translated to an immersed 3D setting. We assessed two primary outcome variables: visual working memory performance and spatial biases in gaze during mnemonic selection after the central color cue. We first confirmed the anticipated patterns of interest in our static reference condition, before turning to evaluating these patterns in our novel (“flow”) condition of interest.

Visual working-memory performance was quantified using three complementary measures: accuracy (quantified as percentage correct), sensitivity (quantified as *d*′), and capacity (quantified as *K*). We added *d*′ and *K* estimates with the primary purpose of forming a bridge to prior literature that used either of these measures, not because we aimed to commit to slot, resource, or other accounts of storage limitations of visual working memory. Indeed, our question regarding the influence of “mode of object disappearance” on performance should be relevant regardless of whether one believes in slots, resources, or otherwise.

Sensitivity, quantified as *d*′, was corrected for the forced-choice setting with six alternatives (DeCarlo, 2012). We used a loglinear approach to correct for extreme proportions such as when participants had all trials correct in a condition (DeCarlo, 2012; Hautus, 1995; Stanislaw & Todorov, 1999).

For completeness, task accuracy was additionally converted to capacity (*K*) scores (Cowan, 2001; Macmillan & Creelman, 2004). For this, we again considered the number of correct target-shaped responses, as well as the number of responses that would be correct by chance, given the number of possible response options (always six in our case). We first calculated the true proportion of incorrect responses by dividing the observed proportion of incorrect trials by 5 and multiplying this by 6 (as one-sixth of guesses would erroneously be classified as “correct” by chance). We then used the true proportion of incorrect responses to obtain the true proportion of correct responses, which we in turn multiplied by load to obtain an estimate of *K*. Consequently, *K* has a ceiling of 2 in load 2 and a ceiling of 4 in load 4. Though measures such as *K* may invite particular theoretical frameworks and come with their own limitations (Brady, Robinson, Williams, & Wixted, 2023; Williams, Robinson, Schurgin, Wixted, & Brady, 2022), we here mainly used *K* to be able to relate our findings to relevant prior work that also reported *K*. Task accuracy was compared between load conditions using two-sided, paired-sample *t*-tests with an alpha level of 0.05. As a measure of effect size, we used Cohen's *d*.

In addition to task performance, our second central outcome variable involved spatial biases in gaze during mnemonic selection after the color cue (Draschkow et al., 2022; van Ede, Chekroud, & Nobre, 2019; van Ede et al., 2020). To this end, we epoched our eye-tracking data around the cue and converted the data into degrees of visual angle (relative to gazing at the central fixation cross). The gaze data were baseline corrected by subtracting average gaze position in the 250-ms time window before cue onset. As part of the gaze analysis, the data were separated by cued object location. Gaze traces were analyzed separately for cues directing attention to memorized objects at any of the 10 possible stimulus configurational positions. Trends in the gaze data that were common among the 10 possible positions were removed to facilitate visualization of the location-dependent biases in gaze. Note that “location” here was defined as the configurational position associated with the cued memory object, even though nothing was presented nor expected at this location during or after the cue.

We visualized the data first by plotting average gaze position for each of the 10 configurational positions of the cued memory object, separately for consequent time frames (bins) of respectively 0 to 500 ms, 500 to 1000 ms, and 1000 to 1500 ms from cue onset. Complementary to this visualization, we also plotted the full time courses of the horizontal gaze position for all 10 configurational positions. Finally, to facilitate quantification and maximize sensitivity, we expressed the spatial gaze bias by a single measure of “towardness” (Draschkow et al., 2022; van Ede, Chekroud, & Nobre, 2019) by considering for each location how much gaze was biased toward this location versus toward the opposite location. To this end, we considered the Euclidean distance of gaze relative to the location of the cued versus the opposite memory object and subtracted the two. We did this separately for each time sample, yielding a single time course of towardness per cued object location (that we then averaged across all 10 possible locations).

Gaze-towardness time courses were statistically evaluated against zero (no spatial bias) using cluster-based permutation analyses (Maris & Oostenveld, 2007). This allowed us to bypass the multiple-comparisons problem by evaluating clusters of neighboring time points (that each exceeded univariate significance levels at an alpha of 0.05) under a single permutation distribution of the largest temporal clusters that would be observed by chance (i.e., after random permutation). We ran this analysis in the FieldTrip analysis toolbox (Oostenveld, Fries, Maris, & Schoffelen, 2011) using 10,000 permutations.

## Results

Twenty-five human volunteers performed a visual working memory task in VR (Figure 2), with two distinct modes of object disappearance. Our static condition mimicked traditional laboratory tasks in which visual objects were held static in front of the participant before being removed from view abruptly. In contrast, in our critical flow condition, the same visual objects were set in motion and flew by participants, thereby disappearing from view gradually in a more naturalistic manner and eventually residing behind the observer.

Below, we first establish the relevant data pattern of interest from the static condition that served as our reference. Thereafter, we evaluate these patterns in our critical flow condition of interest.

### Static reference condition with abrupt object disappearance in front of the participant

**Figure 3.**
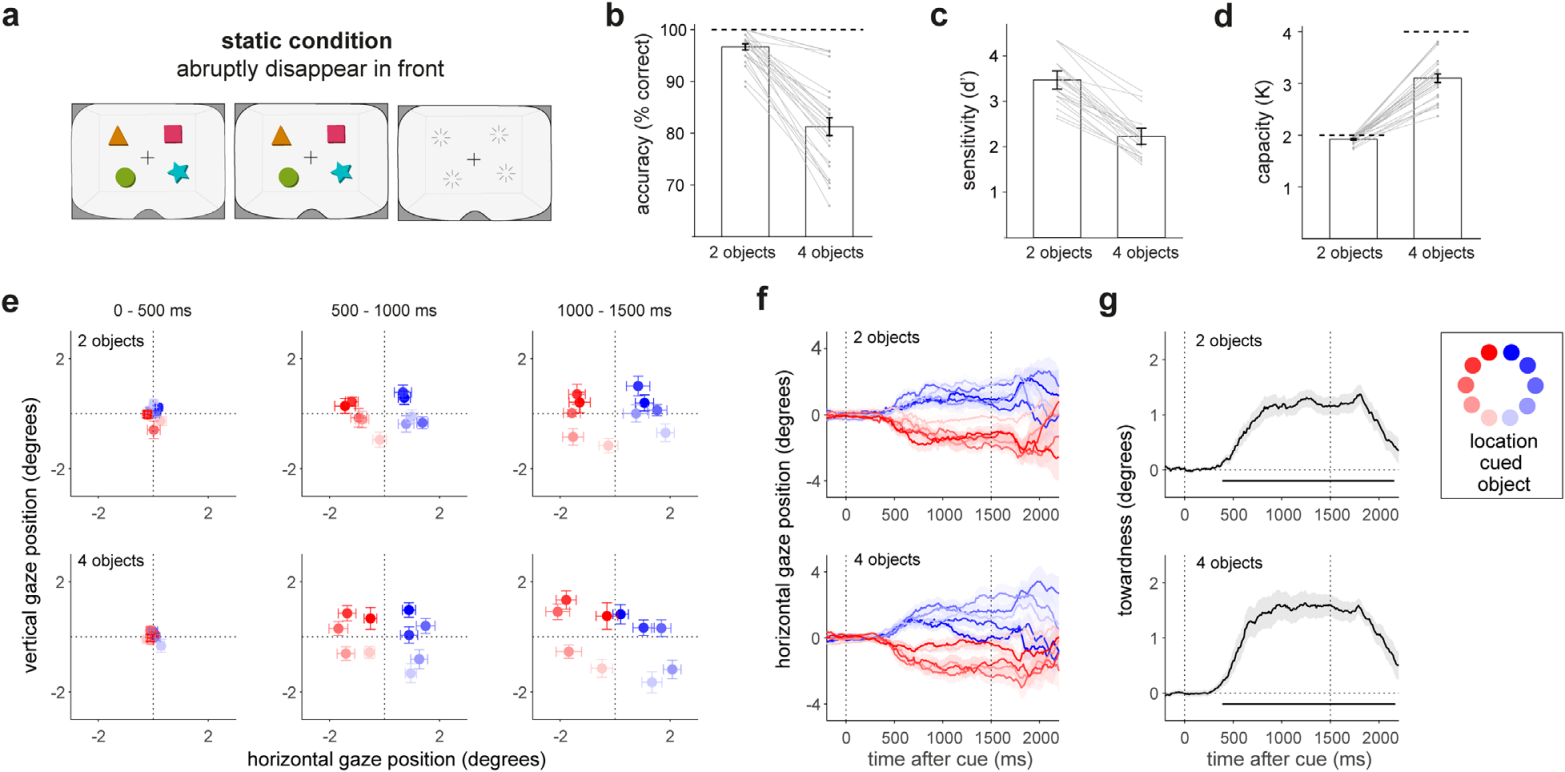
Performance and gaze results from the static condition in which objects disappeared abruptly in front of the participant. (**a**) Data from our static reference condition in which objects disappeared abruptly in front of the observer. (**b**) Recall accuracy (of the cued object) was higher when memory arrays contained two versus four objects, with performance being close to ceiling in load 2 but not in load 4. (**c**) Working memory performance sensitivity estimated as *d*′. (**d**) Visual working memory capacity estimated as *K* paralleled the recall data in panel b. Note how maximum capacity in load 2 was limited to 2, as there were only two objects available to retain in memory. Capacity in our load 4 condition revealed an average visual working memory capacity of around three objects, in line with prior studies using 2D displays and comparable colored shape stimuli. (**e**) Following the color cue, gaze position diverged depending on the encoded location of the cued object (see color code) in both load 2 (top) and load 4 (bottom). (**f**) Time courses of horizontal gaze position showed a clear distinction between cued objects that were presented left of fixation (red) and those right of fixation (blue). (**g**) Towardness bias in gaze in the direction of the cued object, aggregated over all object locations. Horizontal black lines denote clusters of significant towardness (cluster-based permutation analysis).

In our static condition (Figure 3a), visual objects were held static in front of the participant and were removed from view abruptly. This mimics classic laboratory tasks of visual working memory, inside our VR environment. Figure 3b shows the performance accuracy in our static condition. Not surprisingly, we found a robust effect of memory load, *t*(24) = 10.660, *p* = 1.393e-10, *d* = 2.132, with close-to-ceiling performance in load 2 but not in load 4 (Figure 3b), consistent with a capacity above two but below four objects. Likewise, when calculating sensitivity, we found higher *d*′ values for load 2 (*M* = 3.468, *SE* = 0.101) than load 4 (*M* = 2.228, *SE* = 0.09). With the aim to form a bridge to a large literature of prior studies, we also estimated working-memory capacity. We transformed our accuracy scores to capacity scores (denoted *K*) by considering the maximum capacity (given the number of visual objects in the memory display) and the number of correct responses that would occur through guessing (given the number of objects to choose from at the probe stage). Like accuracy, capacity (Figure 3d) was close to ceiling in load 2, with a *K* of 1.920 ± 0.014 (*M* ± *SE*). In the more demanding load 4 condition, in which capacity was further from ceiling, we observed a *K* of 3.100 ± 0.083. This observed *K* around three reveals that the capacity estimates in our VR task are largely consistent with seminal prior work using similar-colored shape stimuli in 2D displays (e.g., Luck & Vogel, 1997; Vogel & Machizawa, 2004).

In addition to memory capacity, we were interested in the incidental use of space for mnemonic selection. To track such space-based mnemonic selection, we capitalized on our recent demonstrations of reliable directional biases in gaze when participants select visual objects within working memory (Draschkow et al., 2022; van Ede, Chekroud, & Nobre, 2019; van Ede et al., 2020; van Ede et al., 2021). As shown in Figures 3e to 3g, in our static condition we confirmed clear gaze biases that are comparable to these studies. Following the color change of the central fixation cross at time 0, gaze position gradually became biased toward (i.e., in the direction of) the memorized location of the cued visual memorandum (Figure 3e). This is also appreciated by the time courses of horizontal gaze position in Figure 3f. When the cued memory object occupied a location on the left during encoding (red traces), gaze became biased to the left, whereas when the cued memoranda was on the right during encoding (blue traces) gaze became biased to the right after the selection cue. To facilitate further evaluation of this spatial gaze bias, we reduced the gaze bias to a single measure of “towardness” (as in Draschkow et al., 2022; van Ede, Chekroud, & Nobre, 2019; van Ede et al., 2020; van Ede et al., 2021). As shown in Figure 3g, this confirmed that our gaze index of space-based mnemonic selection was highly robust—both when the memory load was two (top row; cluster *p* < 0.001) and when the memory load was four (second row; cluster *p* < 0.001).

These gaze biases occurred despite the object location never being asked about and despite there being nothing to see at these locations, nor anything expected at these locations in the interval after the cue (response options always appeared 1500 ms after cue onset and appeared below fixation with the target object randomly positioned in one out of six possible locations). Together, these data show how our VR task captures well-established properties of capacity estimates of visual working memory and the use of space for the selection of objects within visual working memory. Having established this, we next turn to our key flow condition of interest.

### Flow condition with gradual object disappearance behind the participant

**Figure 4.**
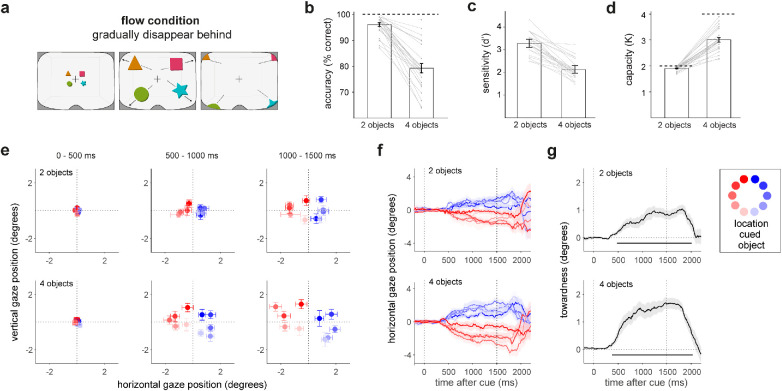
Performance and gaze results from the flow condition in which objects disappeared gradually behind the participant. (**a**) Data from the flow condition in which objects disappeared gradually behind the observer. (**b**–**d**) Recall accuracy, sensitivity, and visual working memory capacity in the flow conditions. Conventions are as in Figure 3. (**e**–**g**) Gaze towardness bias following the cue in the flow condition. Conventions are as in Figure 3.

The key novel condition in our experiment involved the flow condition in which objects gradually disappeared behind observers (Figure 4a). This raised two questions. First, will we still find comparable accuracy, sensitivity, and capacity? Second, will people still rely on space for mnemonic selection after visual objects have disappeared *behind* the observer?

Figure 4 shows the relevant data from this condition (following the same organization as Figure 3). As in the static condition, performance in the flow condition showed a clear effect of memory load, *t*(24) = 10.2895, *p* = 2.807e-10, *d* = 2.807, with close-to-ceiling performance in load 2 but not in load 4 (Figure 4b). This was again also evident in sensitivity, with higher *d*′ values for load 2 (*M* = 3.284, *SE* = 0.092) than load 4 (*M* = 2.127, *SE* = 0.082). Finally, the capacity estimate (Figure 4d) was again close to ceiling in load 2 with a *K* of 1.904 ± 0.017, and capacity in load 4 was 3.003 ± 0.086. These *d*′ and capacity estimates were very similar to what we had observed in the static condition and are again in agreement with a vast body of more traditional research using 2D displays.

We next turned to the gaze data. Having confirmed the spatial gaze bias in our default static condition—in which objects disappeared in front of the participant—we asked what would happen in the critical flow condition where objects ultimately disappeared behind the participant. It is conceivable that in this condition we would no longer find the gaze bias, as space (and the oculomotor system) may no longer be used for mnemonic selection when memory objects reside behind the observer.

As shown in Figures 4e to 4g, despite objects disappearing behind the observer, we still found robust directional gaze biases in our flow condition. Gaze biases were again highly robust, both when load was two (top row; cluster *p* < 0.001) and when the load was four (bottom row; cluster *p* < 0.001).

## Discussion

We set out to investigate whether the mode of visual object disappearance may affect how we store and later select visual objects in working memory. We found that two classic findings from laboratory tasks with static 2D displays—a capacity estimate of around three and the use of space for object selection—generalized to our more naturalistic condition in which objects disappeared gradually behind the observer. We discuss both findings in turn.

### Comparable capacity estimates of visual working memory despite distinct modes of object disappearance

Despite profound differences in mode of object disappearance between our static and flow conditions, we found highly comparable estimates of visual working memory capacity, across both tested memory loads. This is important, as it demonstrates that classic capacity estimates from laboratory tasks in which static visual objects are removed abruptly from view are not restricted to such settings.

At the same time, our results should not be taken as evidence that visual working memory capacity is a fixed quantity. For example, capacity estimates have been found to be higher for real-world and meaningful objects (Asp et al., 2021; Brady et al., 2016) and lower for more complex objects (Alvarez & Cavanagh, 2004; Chen, van Ede, & Kuo, 2022). Moreover, in everyday life, we may not always use our maximum capacity because working memory use can depend on the precise behavioral demands of the task (Ballard, Hayhoe, Pook, & Rao, 1997; Draschkow et al., 2021; Kristjánsson & Draschkow, 2021; Somai, Schut, & van der Stigchel, 2020). Here, we deliberately used simple colored shapes in a simple working memory task to mimic seminal work by ample laboratory studies before us (e.g., Luck & Vogel, 1997; Vogel & Machizawa, 2004). Doing so, we were able to confirm a capacity estimate of around 3 using such shapes also in our static VR condition, allowing a critical bridge to a long tradition of laboratory studies before us that used 2D displays (for a complementary comparison of 2D vs. 3D objects, see also He, Gunalp, Meyerhoff, Rathbun, Stieff, Franconeri, & Hegarty, 2022). Having established that bridge, we asked whether the capacity estimate observed with this type of visual shapes and task may be different when objects disappear from view gradually in a more naturalistic manner. We found no evidence for this. This is reassuring and important (in light of the increasingly appreciated “generalizability crisis”) (Yarkoni, 2022), because it suggests that capacity estimated in more artificial tasks with abrupt object disappearance may generalize to more naturalistic modes of object disappearance.

### Comparable space-based mnemonic selection when objects disappear in front of or behind the observer

Besides performance and capacity, we were interested in whether participants would continue to rely on spatial coding strategies to individuate and later select memory contents when these contents entered working memory after disappearing behind the observer. Here, the use of space may break down, provided the memorized object locations themselves are out of view behind the observer. To address this, we tracked spatial biases in gaze that we previously linked to attentional focusing to objects within visual working memory (de Vries et al., 2023; Draschkow et al., 2022; van Ede, Chekroud, & Nobre, 2019; van Ede et al., 2020; van Ede et al., 2021) even when memorized object locations are never asked about (as was also the case in the current task).

Strikingly, we observed a gaze bias not only when visual objects were remembered in front of the participant (replicating our prior studies), but also when they had disappeared behind the observer. Given the similarities in the gaze biases between our static and flow conditions, our data suggest that similar spatial coding may be utilized for mnemonic selection of objects, irrespective of their current location relative to the observer (i.e., in front or behind). This may possibly reflect a strategy whereby participants imagine a “snapshot” of the objects in front of them, even when they disappeared behind them. Alternatively, it is possible that the oculomotor system may be engaged for covert (here internal) selective attention, even for visual targets that are outside the oculomotor range (see, for example, Hanning, Szinte, & Deubel, 2019; Tark & Curtis, 2009). Either way, our data suggest that participants retain the configuration of the objects and use this for mnemonic selection—as read out from their gaze. In future studies it could be interesting to vary the visual flow of objects in such a way that it would also be possible to disentangle multiple frames of references (e.g., allocentric and egocentric) and investigate which spatial frames are used for selection (cf. Draschkow et al., 2022; Heuer & Rolfs, 2022; Klinghammer, Schütz, Blohm, & Fiehler, 2016; Pertzov, Avidan, & Zohary, 2011) following naturalistic object disappearance in such a context.

What exactly the gaze bias reflects remains an interesting question. We propose that it at least reflects spatial orienting of attention within the spatial layout of working memory. At the same time, it may be sensitive to additional processes, such as the amount of resources required to shift attention or the resources devoted to the object after attention has already shifted. Such accounts may explain why the gaze bias appears larger with higher memory load (as also reported in Draschkow et al., 2022), although we cannot exclude alternative explanations. For example, with lower load, participants may be more likely to verbalize the visual objects and therefore rely less on memorized visual locations during retention and subsequent mnemonic selection.

Another interesting question regards the behavioral relevance of this bias. We have previously linked this type of gaze bias during mnemonic selection to behavioral accuracy in two studies. In one study, we found that a larger gaze bias to the correct memory item predicted working-memory accuracy, particularly when the task was more demanding (i.e., following anti-cues; see Figure 4a in van Ede et al., 2020). In another study, where we specifically focused on microsaccades, we found that memory reports were more accurate in trials with toward vs. away microsaccades (see Figure 2a in Liu, Nobre, & van Ede, 2022). We were eager to also look at this in our current VR dataset, separately for our static and flow conditions and for load 2 and load 4. Unfortunately, however, because we used a large set of possible stimulus locations (*n* = 10), we ran into a logistic problem of not having sufficient incorrect trials for several target-location conditions that were required to calculate our towardness gaze-bias metric.

### Future directions

Our approach provides an important step forward within the larger movement to study visual working memory under more naturalistic (Brady et al., 2016; Draschkow et al., 2021; Klinghammer et al., 2016; Kristjánsson & Draschkow, 2021; Li, Aivar, Kit, Tong, & Hayhoe, 2016; Snow & Culham, 2021; Tatler, Hirose, Finnegan, Pievilainen, Kirtley, & Kennedy, 2013; Tatler & Land, 2011) and less static (Chung, Schurgin, & Brady, 2023; Heuer & Rolfs, 2021b) conditions. At the same time, there are a number of points that call for future experiments.

First, we used simple colored shapes that were floating in an otherwise empty room. As elaborated above, we deliberately did this to provide a direct bridge to traditional laboratory tasks with 2D displays. Doing so, we were successful at replicating key findings from such tasks in our static VR setting, which served as an important reference for evaluating our flow condition of interest. Still, in future studies, it would be interesting to additionally manipulate the realistic nature of the objects and the surroundings they are embedded in.

Second, we simulated optic flow by having the objects move while the observer remained still. In future studies, it would be interesting to simulate a condition in which the observer moves or where both the observer and the environment are in motion.

Third, although we focused on mode of object disappearance, our flow and static conditions also differed in other aspects. For example, only in our flow conditions were objects moving. Object motion by itself may have negatively affected sensory encoding and memory consolidation (Wolbers, Hegarty, Büchel, & Loomis, 2008) and/or may have become an additional feature in memory (Shen, Gao, Ding, Zhou, & Huang, 2014). We cannot rule out that potential detrimental consequences of the motion itself may have canceled out potential benefits of more naturalistic modes object disappearance.

Finally, although we found no evidence for obvious qualitative differences by mode of object disappearance, we cannot rule out that more subtle differences may exist or that relevant differences may exist for memory aspects other than capacity and selection. Therefore, in future studies, other things could be investigated as a function of mode of object disappearance, such as susceptibility to subsequent interference (Gresch, Boettcher, van Ede, & Nobre, 2021; Lorenc, Mallett, & Lewis-Peacock, 2021) or the concurrent engagement of action planning alongside visual working memory (Boettcher, Gresch, Nobre, & van Ede, 2021; Nasrawi & van Ede, 2022; Schneider, Barth, & Wascher, 2017; van Ede, Chekroud, Stokes, et al., 2019). These and possibly other aspects may still show qualitative differences when visual objects disappear abruptly versus gradually and/or in front versus behind the observer.

Future possibilities aside, the present study provides a first step toward studying visual working memory capacity and selection following more naturalistic modes of object disappearance. This first step has brought the reassuring insight that at least certain classic findings from traditional (static and 2D) laboratory tasks of visual working memory may generalize to more naturalistic modes of object disappearance.
